# 3D magnetization transfer (MT) for the visualization of cardiac free-running Purkinje fibers: an ex vivo proof of concept

**DOI:** 10.1007/s10334-020-00905-w

**Published:** 2021-01-23

**Authors:** Julie Magat, Arnaud Fouillet, Marion Constantin, Kylian Haliot, Jérôme Naulin, Dounia El Hamrani, David Benoist, Sabine Charron, Richard Walton, Olivier Bernus, Bruno Quesson

**Affiliations:** 1 IHU Liryc, Electrophysiology and Heart Modeling Institute, Foundation Bordeaux Université, Hopital Xavier Arnozan, Avenue du Haut Lévêque, 33604 Pessac cedex, France; 2 Centre de Recherche Cardio-Thoracique de Bordeaux Inserm, U1045, Université de Bordeaux, 33000 Bordeaux, France

**Keywords:** Magnetization transfer, Cardiac conduction system, Free-running Purkinje fibers

## Abstract

**Objectives:**

We investigate the possibility to exploit high-field MRI to acquire 3D images of Purkinje network which plays a crucial role in cardiac function. Since Purkinje fibers (PF) have a distinct cellular structure and are surrounded by connective tissue, we investigated conventional contrast mechanisms along with the magnetization transfer (MT) imaging technique to improve image contrast between ventricular structures of differing macromolecular content.

**Methods:**

Three fixed porcine ventricular samples were used with free-running PFs on the endocardium. *T*1, *T*2*, *T*2, and *M*0 were evaluated on 2D slices for each sample at 9.4 T. MT parameters were optimized using hard pulses with different amplitudes, offset frequencies and durations. The cardiac structure was assessed through 2D and 3D *T*1w images with isotropic resolutions of 150 µm. Histology, immunofluorescence, and qPCR were performed to analyze collagen contents of cardiac tissue and PF.

**Results:**

An MT preparation module of 350 ms duration inserted into the sequence with a *B*1 = 10 µT and frequency offset = 3000 Hz showed the best contrast, approximately 0.4 between PFs and myocardium. Magnetization transfer ratio (MTR) appeared higher in the cardiac tissue (MTR = 44.7 ± 3.5%) than in the PFs (MTR = 25.2 ± 6.3%).

**Discussion:**

MT significantly improves contrast between PFs and ventricular myocardium and appears promising for imaging the 3D architecture of the Purkinje network.

**Supplementary Information:**

The online version contains supplementary material available at 10.1007/s10334-020-00905-w.

## Introduction

The specialized cardiac conduction system (CCS) in the ventricles enables rapid and near synchronous excitation of the ventricular myocardium. It is composed of an extensive branching network of Purkinje fibers (PF) ensuring rapid electrical conduction due to specific electrical properties and a fibrous structure surrounded by a collagen sheath [1, 2]. These PF differ from working myocardial cells in size, function, structure, and arrangement [3]. The Purkinje conduction network plays a crucial role in normal cardiac function, but it has also been implicated in arrhythmogenesis and sudden cardiac death [4, 5]. 3D imaging of its architecture is, therefore, of high importance to better understand cardiac arrhythmia mechanisms and improve their diagnosis and treatment.

The most common experimental techniques to study the architecture of the cardiac electrical conduction system are based on destructive approaches such as histology [6]. Ink injections have also been used to follow PF ramification in sheep until their termination within the myocardium, but do not allow for quantitative reconstructions [7]. Recently, μ-computed tomography has been applied with a very high resolution of 18 µm on rat heart [8] to follow the CCS. The same team published a 3D representation of the CCS in the intact human heart with an isotropic resolution of 73 µm [9]. However, disadvantages of the X-ray-based techniques are the ionizing radiation, the ability of contrast enhancement to discriminate between different tissues is limited and the transfer of method from microCT to medical CT facilities is currently not available.

Alternatively, magnetic resonance imaging (MRI) is another approach for non-destructive 3D acquisition of tissue samples. Some studies have developed algorithms to track free-running PF from high-resolution images in rodent hearts with an isotropic resolution of 25 µm [10–12]. Hwang et al. [13] demonstrated at 17.6 T using a fast gradient echo sequence an alteration with aging in rabbit hearts especially on free-running PF thickness.

Different MRI acquisitions can be exploited to improve contrast in biological tissues, depending on their proton density and relaxation times. However, all these MR approaches suffer from an obvious lack of contrast between CCS and cardiac muscle on ex vivo fixed samples at high field [14].

MT MRI [15] has been widely been used in neuroradiology to visualize myelin defects in multiple sclerosis [16], as well as to suppress background signals for angiography applications [17]. It consists of using a preparation module (generally with off-resonance pulses) that saturates a portion of the proton spectrum from macromolecules and reduces partially the signal from the water proton frequency through transfer of magnetization [18]. MT is more specific for assessing macromolecular contents than classical contrast MRI techniques (*T*1, *T*2 and proton density weighted images). Several approaches using MT for collagen detection have been applied to evaluate fibrosis in liver [19], in kidney [20, 21] and fibrosis in intestinal tissue [22]. However, MT has rarely been investigated for cardiac imaging; and only few studies have used MT in vivo with off-resonance pulses to increase contrast between blood and muscle [15] and between lesion sites (infarct, hypoxia and edema areas) and healthy cardiac tissue [23]. Interestingly, a recent study [24] applied MT preparation in vivo to assess myocardial infarct. They demonstrated a difference of contrast between scar areas and healthy tissues. They found a good correspondence between low MTR and hyperintense signal using LGE for the assessment of myocardial scars.

Several types of collagen (mainly type I and III) were reported to be present in the heart with different expression levels in myocardium and PF [2, 25]. Moreover, different MT ratios were measured depending on the type of collagen [26], making this technique a potential candidate for increasing image contrast between PF and myocardium.

In this work, we first quantitatively analyzed the contrast between PF and cardiac muscle using conventional MRI techniques based on a difference of structure. In a second step, off-resonance MT was applied in 2D to assess MT parameters that optimize the contrast to present a proof of concept to distinguish PF from cardiac tissue. From these results, 3D high-resolution images were acquired with MT with the objective of visualizing the free-running PF in cardiac tissue samples.

## Methods

### Ex vivo samples

Fixed ex vivo cardiac samples (*n* = 3) were obtained from three ~ 40 kg male pigs under general anesthesia. Hearts were removed by sternal thoracotomy and flushed with cold cardioplegic solution. This protocol was accepted by the Animal Research Ethics Committee (Comité d’Ethique en Expérimentation Animale de Bordeaux; CEEA50) in accordance with the European rules for animal experimentation (European legislation 2010/63/UE; 2010).

**Fig. 1:**
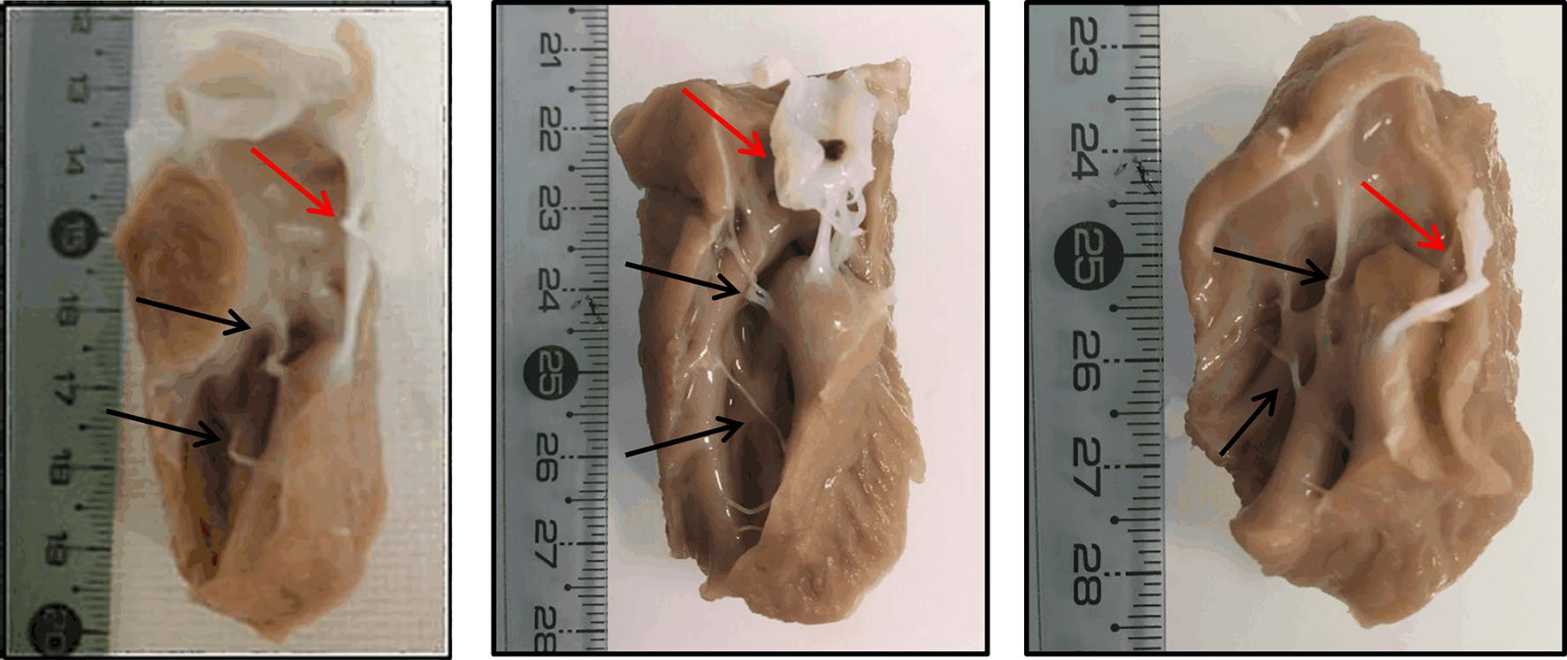
Photographs of three ex vivo samples with contrast agent after fixation. Black arrows indicate free-running fibers of interest. Red arrows indicate tendons connected to valves

Left ventricular samples from the anterior side of the heart were chosen due to the natural abundance of free-running fibers in this region. Samples were cut in the longitudinal direction (Fig. 1). Three samples were prepared immersed in 10% formaldehyde mixed with an MRI contrast agent (0.2% of GD DOTA was added of the total formaldehyde volume): sample 1 with a dimension of 3 × 4 × 7 cm, sample 2 with a dimension of 4 × 4 × 6 cm and sample 3 with a dimension of 3 × 3 × 5 cm. Samples were continuously and mechanically agitated in their fixing solution during 2 days in a cold room and then stored in their fixing solution in the same cold room.

For MRI acquisition, samples were removed from the solution and were immersed into fluorinert (Invertec Performance Medicals, Saint Priest France), a perfluoropolyether, giving no ^1^H MR signal and having similar magnetic susceptibility as tissue [27].

### MRI experiments

#### Magnet

Experiments were performed at 9.4 T within a horizontal bore of 30 cm (Bruker BioSpin MRI, Ettlingen Germany) with BGA-20S gradient system (maximum strength of 300 mT/m, slew rate of 1170 T/m/s). A cylindrical single-coil element coil (83 mm inner diameter, with a maximum of 28 W power deposit in continuous wave) operating in transmission and reception was used for fixed ex vivo imaging. A temperature probe (SA Instruments, Stony Brook, NY) was placed on the top of the samples to record temperature during acquisitions.

#### MRI sequences

2D slice with a resolution of 200 µm × 200 µm × 1 mm using a FOV = 30 × 30 mm and a matrix = 150 × 150 with a bandwidth of 75 kHz was applied for *T*1, *T*2, *T*2* and proton density (*M*0) imaging on samples.

A rapid acquisition with relaxation enhancement (RARE) sequence was used to determine *T*1 relaxation time with a saturation recovery mode, where TR varied to collect series of *T*1 weighted images. Imaging parameters were: TE = 30.6 ms, TR array = 200, 400, 800, 1500, 5000 and 10,000 ms, RARE factor = 8 for a total acquisition time of 5 min 22 s.

A multi-echo spin echo sequence was used to determine *T*2 relaxation time with TR = 2000 ms, NA = 1, 8 echoes starting at 10 ms with increments of 10 ms for a total acquisition time of 5 min.

A multi-gradient echo sequence with eight echos was launched to obtain *T*2* information with TR = 800 ms, NA = 1, 8 echoes starting at 4 ms with increments of 5.5 ms for a total acquisition time of 2 min.

#### 2D magnetization transfer (MT) imaging

For each 2D acquisition, the sequence parameters were: TE/TR = 4/2000 ms; FOV = 30 × 30 mm matrix size = 150 × 150; FA = 90°; NA = 2; a bandwidth of 50 kHz for a total acquisition time of 10 min with a resolution of 200 × 200 × 500 µm. The MT module (hard pulse of amplitude *B*1, offset frequency Δ*f* and module duration *D*) was added to the sequence before the excitation pulse of the FLASH sequence. One pulse lasts 5 ms with a bandwith of 256 Hz. The following parameters were evaluated to study their influence on the contrast between fiber and muscle:

Δ*f*: 2000, 3000, 5000 and 10,000 Hz.

B1: 5, 10 and 15 μT.

*D*: 50, 150, 250, 350, 450 ms.

#### 3D magnetization transfer (MT) imaging

The use of a contrast agent allows to decrease TR and consequently to increase the power deposit. FLASH acquisition using the MT module (*B*1 = 10 µT, *D* = 350 ms and Δ*f* = 3000 Hz) in 3D was acquired with TR = 350 ms, TE = 4.3 ms, NA = 4, FA = 60°, Bandwidth of 50 kHz and a resolution = 150 × 150 × 150 µm^3^. For the first sample, a FOV = 35 × 40 × 35 mm using 466 × 266 × 233 pixels, for a total acquisition time of 24h6 min is applied, for the second sample a FOV = 60 × 40 × 40 mm using 400 × 266 × 266 pixels, for a total acquisition time of 27h 30 min and for the third sample a FOV = 50 × 30 × 30 mm using 328 × 200 × 200 pixels, for a total acquisition time of 15h 30 min is applied.

### Data processing

Images were exported and post-processed using a homemade program written in Matlab. *T*1, *T*2*, *T*2 of tissue and PF were obtained from exponential curve fitting on a pixel-by-pixel basis. Two ROIs localized in the fiber and muscle were manually drawn, from which the mean and standard deviation of relaxation times were evaluated. *M*0 was extracted on *T*2* fitting using a nonlinear fit with three unknown variables.

For each magnitude image sequence, three ROIs were manually drawn: (1) surrounding the free PF, (2) in the tissue (parenchyma) and (3) in a region considered as the background to assess noise. Manually ROIs in the cardiac tissue, fiber, and noise were drawn. Signal-to-noise ratio (SNR) for PF and tissue were computed as a mean of signal for voxels inside the ROI in the tissue of interest (muscle or fiber) divided by as the average standard deviation of voxel intensity obtained in ROI from the background region for the volume coil. A magnetization transfer ratio (MTR) was defined by MTR = 100*(*S*_0 _– *S*_MT_)/*S*_0_.

Where *S*_0_ represents the signal without an MT module and *S*_MT_ is the signal with the MT module. The MT contrast between fiber and tissue was calculated as follow using contrast to noise ratio (CNR) defined by signal difference between fibers and the surrounding parenchyma divided by background noise:

$${\text{CNR}} = {2}\left( {{\text{SNR}}_{f} - {\text{SNR}}_{t} } \right)/\left( {{\text{SNR}}_{f} + {\text{SNR}}_{t} } \right).$$

where SNR_*f*_ and SNR_*t*_ are the SNR of the fibers and parenchyma, respectively. Visualization of 3D volume rendering was obtained using Volview (Kitware, Clifton Park, NY). Free-running PF segmentation was performed using Snake Interaction Mode algorithm in 3D with itksnap software (http://www.itksnap.org).

### Histological analysis

Histological analysis was performed to identify Purkinje fibers.

Volumes of 0.4 × 1.5 × 0.5 cm were obtained from the three samples. Sections were localized around an intact external “free-running” fiber. Each block of tissue was embedded in paraffin and sectioned at 6 µm in the longitudinal fiber axis. Tissue sections were stained with Masson’s trichrome for the identification of different structure: coloration in blue–green reveals collagen, cellular nuclei are stained in blue–black and myocytes in red. Photographs were taken on an optical microscope (Nikon Eclipse 80i, Nikon, Tokyo, Japan) equipped with a digital camera (Nikon Ds Fi2).

### Detection of collagen types I and III

Additional analyses were performed to assess the relative contents of collagen I and III in purkinje fibers and cardiac myocytes, using immunofluorescence staining (qualitative imaging) and Reverse Transcription quantitative PCR (quantitative measure at the mRNA level).

#### Immunofluorescence staining

Frozen sections (10 µm thick) of ventricular myocardium and PFs taken from pig hearts (*N* = 3) were fixed into formalin (10%). Following a blocking step (10% normal goat serum, Uptima-Interchim UP379030), sections were incubated with primary antibodies rabbit polyclonal anti-collagen I (Abcam: catalog no. ab34710; 1:100) or rabbit polyclonal anti-collagen III (Abcam: catalog no. ab37778; 1:100) overnight at 4 °C. Sections were then washed in PBS and incubated during 1 h at room temperature with secondary antibodies Alexa Fluor 488 Chicken anti-rabbit (Invitrogen A21441; 1/200) or Alexa Fluor 555 Donkey anti-rabbit (Invitrogen A31572; 1/200). Nuclei were stained with DAPI (Invitrogen D1306, 1:100,000 in PBS).

Images were captured under an inverted fluorescence microscope (Nikon NiE) with DSRi2 camera (Nikon).

#### Reverse transcription quantitative PCR (RT-qPCR)

Samples (ventricular myocardium and PF) from three pig hearts were dissected out and immediately placed into RNA later Buffer. Biopsies were then stored at 4 °C until RNA extraction. Total RNA was extracted from tissues using QIAzol reagent (QIAGEN). RNA was purified and DNase treated using the QIAGEN RNeasy Kit (QIAGEN). RNA quantity was assessed by spectrophotometry (NanoDrop/Thermofisher).

Sequences for primers were obtained from Ensembl Genome Browser. Primers were designed using Primer designing tool (NCBI) and synthesized at Sigma Aldrich/Merck. 400 ng of RNA was reversed transcribed using a cDNA Reverse Transcription kit (BIO-RAD) according to the manufacturer’s protocol. RT-qPCR was performed in a 10-μL reaction volume (1 μL cDNA, 5 μL of SYBR Green mix (BIO-RAD), a volume with a concentration of 10 µm upstream and downstream primers respectively, and added ddH2O to 10 μL on the BIO-RAD C100 Touch Thermal Cycler/CFX96 Real-time System. Expression levels for Col1a1 (collagen type I) and Col3a1 (collagen type III) were normalized using a normalization factor calculated by CFX Manager software (BIO-RAD) and based on RT-qPCR results for two selected housekeeping genes, HPR*T*1 and GUSB.

#### Statistical analysis of qPCR

Data are presented as mean ± SEM for three pigs. Two non-parametric tests were used (2 *t* test/Mann–Whitney): one to compare the response to collagen I between cardiac tissue and PF and one to compare the expression of collagen III between muscle and fiber. Samples were considered significantly different for p values lower than 0.05. One asterisk (*) identifies *P* values between 0.01 and 0.05.

## Results

### MR parameters

The mean relaxation times (*T*1, *T*2 and *T*2* in milliseconds) and mean of *M*0 (a.u) are listed in Table 1. *T*1 in the fiber is equal to 458 ± 42 ms and is quite similar with *T*1 in the tissue (428 ± 21 ms). *T*2 exhibits values to 16.0 ± 1.8 ms and 18.1 ± 0.4 ms in the tissue and the fiber, respectively. Note that *T*2 was smaller in the tissue whereas *T*2* values were similar in the muscle and the fiber for all samples with values closed to 11.5 ms. Signals from *M*0 appeared to be closed in the tissue and the fiber for all samples: *M*0 = 31.6 ± 3.9 a.u. and *M*0 = 31.1 ± 3.2 a.u. for cardiac tissue and fiber, respectively. From these data, very limited contrast can be obtained between fiber and tissue using conventional imaging techniques, justifying the need for alternative contrast enhancing approaches. Theoretically, no contrast can be evaluated for using FLASH sequence between fiber and cardiac tissue with these relaxations times and *M*0 values.

**Table 1:** Mean of MR parameters *T*1, *T*2 and *T*2* (ms) are calculated and *M*0 (a.u) measured for both cardiac tissue and fiber

	Tissue	Fiber
T1 (ms)	458±42	428±21
T2 (ms)	16.0±1.8	18.1±0.4
T2* (ms)	11.3±2.5	11.6±2.1
M0 (a.u.)	31.6±3.9	31.1±3.2

### MT optimization

**Fig. 2:**
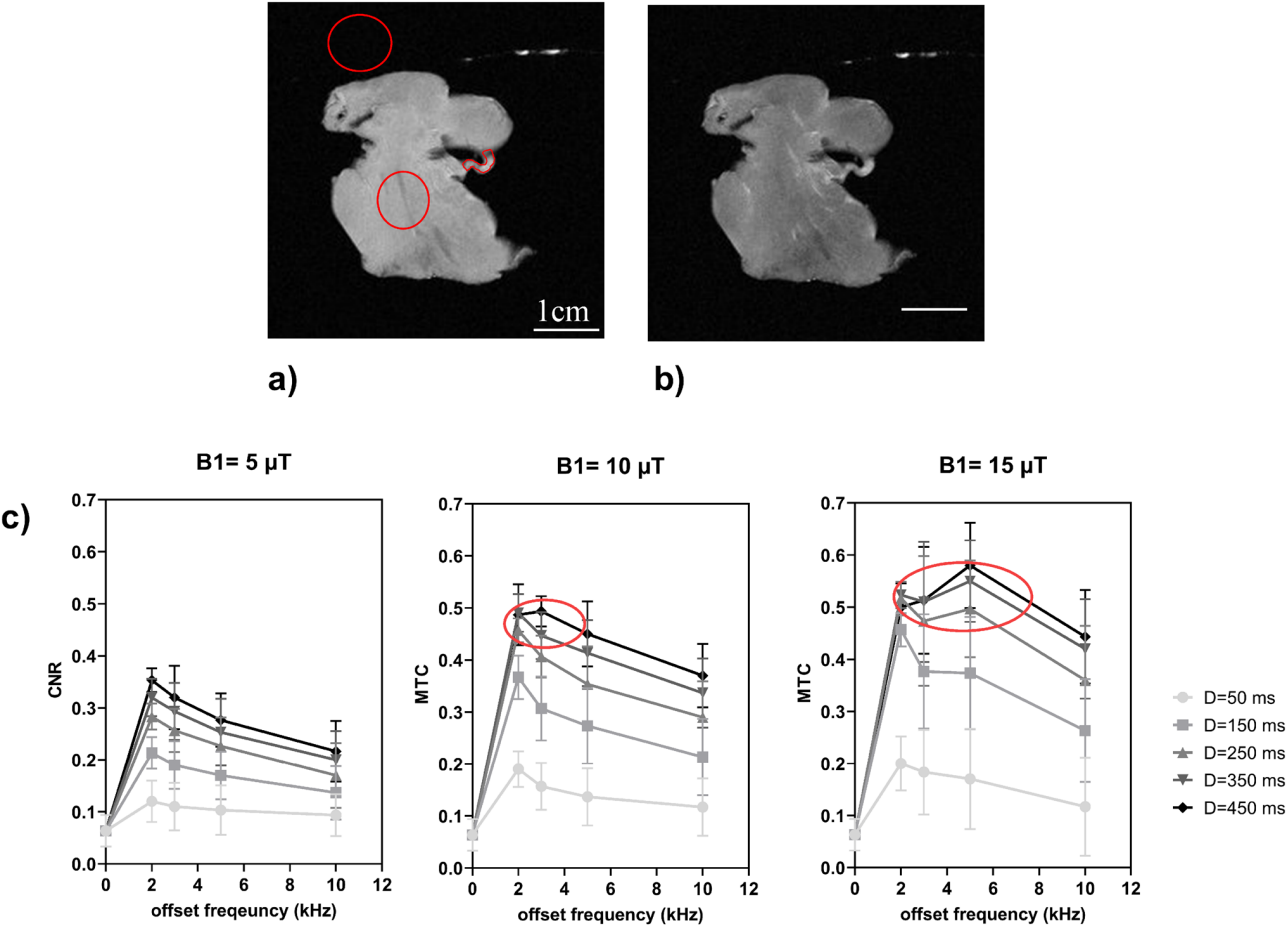
2D MR images without (**a**) and with (**b**) MT (Δ*f* = 5 kHz, *B*1 = 10 μT, *D* = 350 ms). **c** Comparison of contrast of magnetization (CNR) in function of the offset frequency (2, 3, 5 and 10 kHz) at various *B*1 amplitude (5, 10, 15 μT) and MT module duration (50, 150, 250, 350 and 450 ms). The contrast without MT is displayed at zero offset frequency. Maximal values for the contrast between tissue and fibers were found at frequency offset of 3 kHz, for amplitude of 10 μT applied during 350 ms and at frequency offset of 5 kHz, for amplitude of 15 μT applied during 450 ms (red ellipses)

Without MT (Fig. 2a), a nearly homogeneous signal intensity was observed (proton density weighted sequence) for the first sample. In presence of MT, the contrast is improved and more structural details can be identified (Fig. 2b).

 CNR was calculated for each sample using a region of interest in the tissue and a region of interest along fibers (red region of interests in Fig. 2a). Graphs on Fig. 2 represents CNR mean for cardiac samples without and with MT as function of Δf (2, 3, 5 and 10 kHz), *B*1 (5, 10, 15 μT) and D (50, 150, 250, 350 and 450 ms).

The contrast value without MT (equal to 0.06 ± 0.03) is plotted at a zero offset frequency on each graph. Whatever the MT pulse parameters, contrast was systematically higher with the preparation module. However, contrast curves show different values depending on the MT parameters: maximal values for the contrast between tissue and fibers were found at a frequency offset of 2–4 kHz (red ellipses on Fig. 2c), for an amplitude of 10 μT applied during 350 ms (maximal value of 0.52) or for an amplitude of 15 μT applied during 450 ms (maximal value of 0.65).

**Fig. 3:**
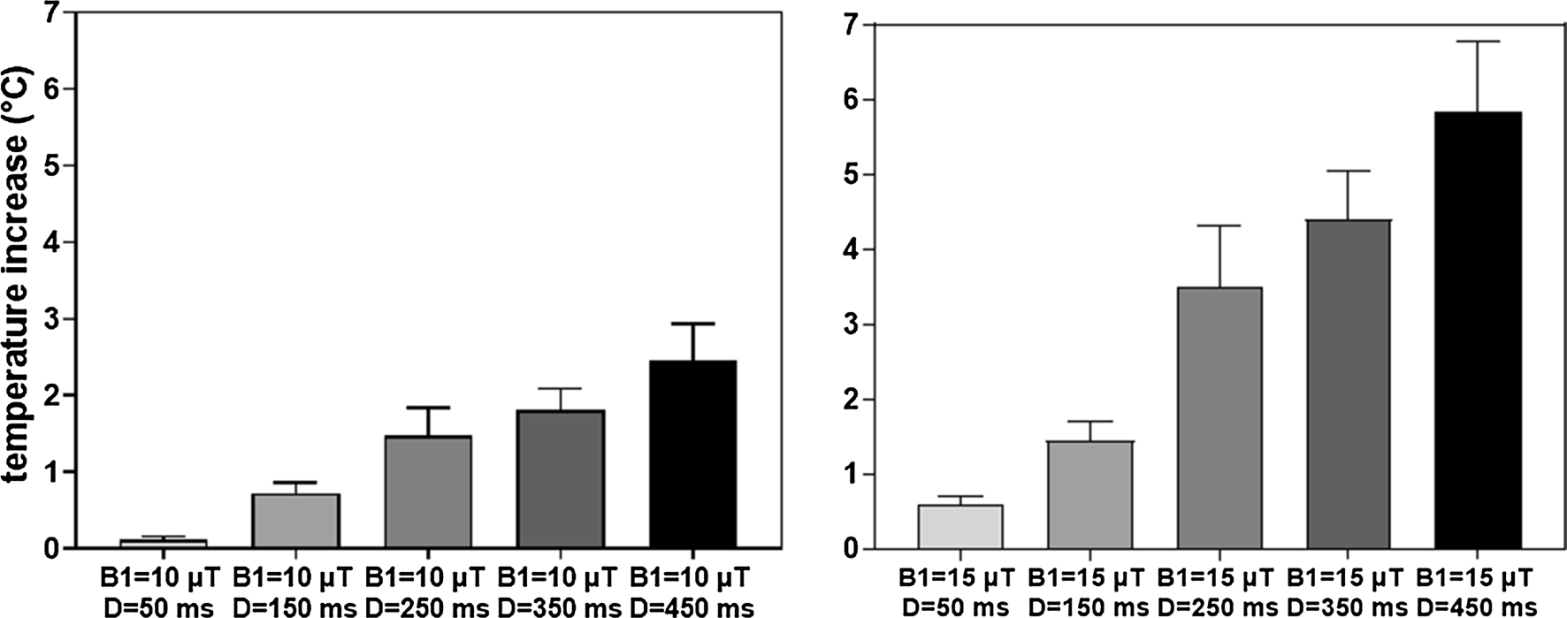
Maximal temperatures raise measured during 2D acquisition with MT modules applied. Histograms represent temperature as a function of *B*1 = 10 μT w for the first graph and *B*1 = 15 μT for the second graph combined with MT module duration of 50, 150, 250, 350 and 450 ms

For *B*1 = 10 µT and *B*1 = 15 µT, temperature was monitored with a raise of temperature during 2 min and a plateau of maximal value until the end of acquisitions. The mean of the temperatures during the plateau is reported in Fig. 3 during 2D acquisition with MT module as a function of *B*1 (10 and 15 μT) and D (50, 150, 250, 350 and 450 ms) at the same repetition time TR = 2 s. No temperature increase is measured for *B*1 = 5 µT at different module duration. Histograms present an increase of temperature below 2 °C for *B*1 = 10µT and *D* ≤ 350 ms. When *B*1 = 15 µT and *D* ≥ 350 ms, the temperature is above 4 °C and reaches a maximum value of 5.8 °C with *B*1 = 15 µT and *D* = 450 ms. MT parameters is chosen with *B*1 = 10 µT, *D* = 350 ms and Δ*f* = 3 kHz with minimum effect of temperature (less than 2 °C) and good contrast between fiber and cardiac tissue for 2D acquisitions.

### MT characterization

**Fig. 4:**
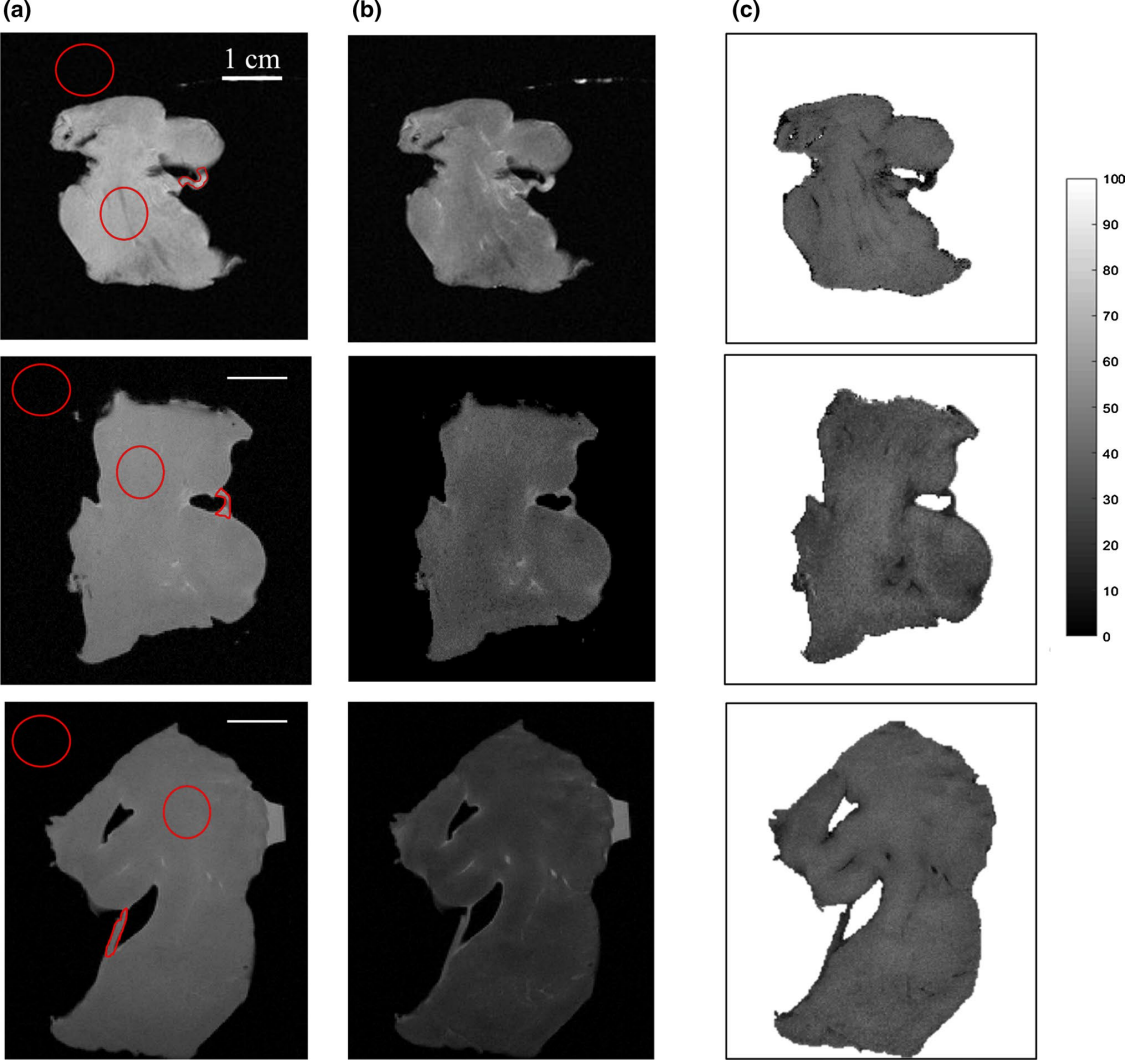
2D images obtained **a** without the preparation module and **b** using MT for sample 1 (first line), sample 2 (second line) and sample 3 (third line). **c** MTR ratio is evaluated between 0 and 100%

Figure 4 displays 2D MR images without MT module, with MT module and MTR maps (Fig. 4a–c) for sample 1 (first line) sample 2 (second line) and sample 3 (third line). Figure 4b shows that the signal is greater for the external fiber and several discrete structures within the muscle, whereas the bulk of the myocardium showed an attenuation of signal. MT effects are visualized by the MT ratio in Fig. 4c, for the fiber the MTR appears to lower than in the tissue.

Table 2 displays mean of SNR and mean of MTR from the fiber and the tissue for all three samples (extracted for regions of interest defined by red ROI on Fig. 4a), together with mean CNR. Under these conditions, SNR from tissue and fibers without MT preparation are similar, reaching approximately 71.6 ± 18.4 and 77.2 ± 18.6. We notice that the SNR in the fiber is always higher (45% of increase) than in the cardiac muscle with a MT module. Moreover, MT is more effective in the muscle: MTR = 44.7% in the tissue whereas MTR = 25.2% for the fiber (see Table 2). Nevertheless, the CNR between myocardium and fibers calculated is studied with a contrast close to 0.38 in mean for all samples.

**Table 2:** Mean of SNR, CNR, and MTR (%) on 2D image slices of the tissue and fiber under the same acquisition parameters

	SNR fiber	SNR tissue	CNR	MTR fiber (%)	MTR tissue (%)
Without MT	77.2±18.6	71.6±18.4	0.06±0.03	–	–
MT	58.4±16.5	40.1±12.4	0.38±0.8	25.2±6.3	44.7±3.5

### 3D MT visualization

**Fig. 5:**
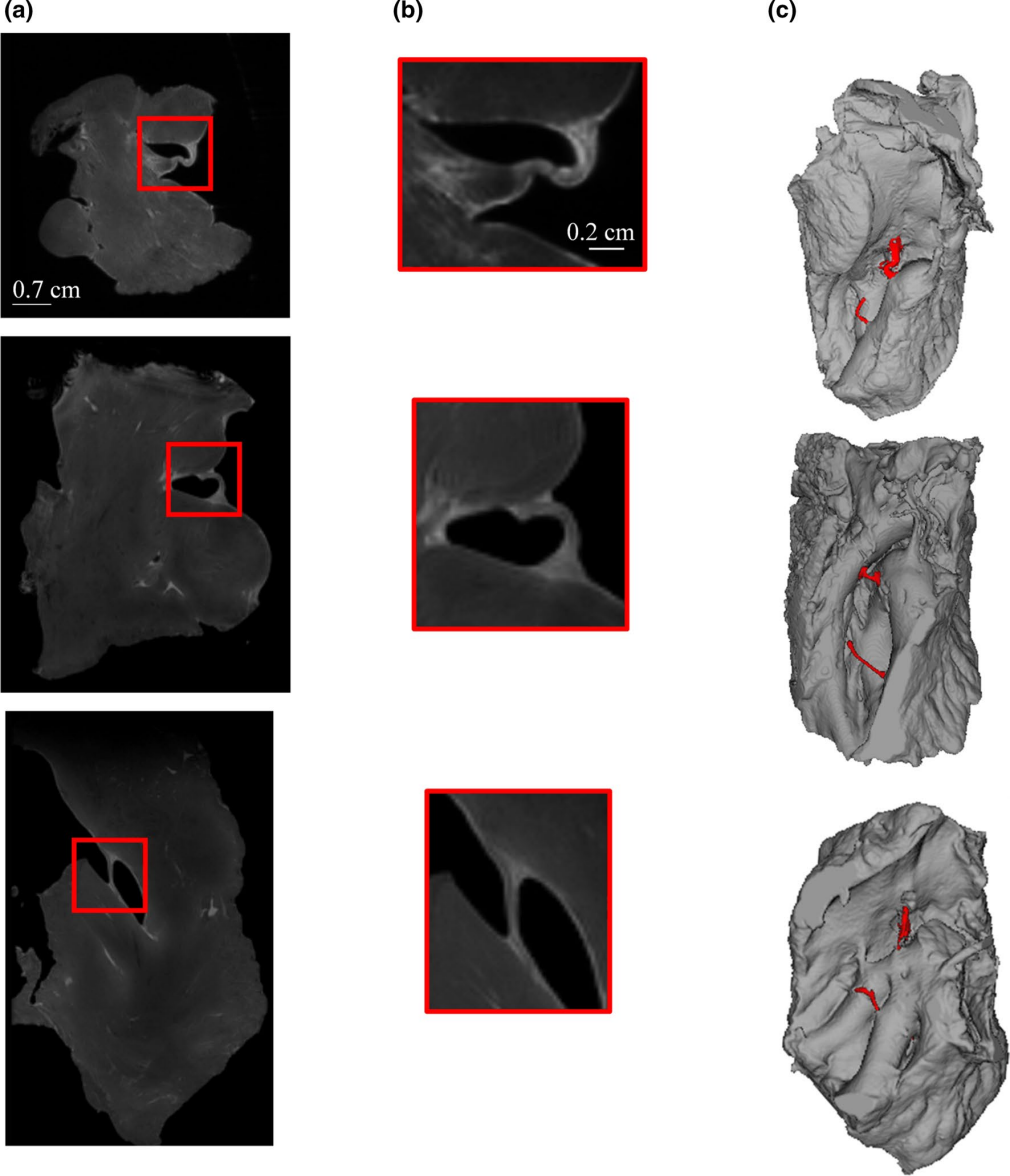
**a** Transverse slices orientated from the 3D volume at an isotropic resolution of 150 μm using MT MRI for sample 1, 2 and 3. Insertion points of the fiber into the myocardium are expanded in the red box and zoomed (**b**). **c** Fibers are segmented in red on a 3D volume rendering using ItkSnap

Figure 5 presents 2D views extracted from 3D acquisition with TR = 350 ms for the three samples with identical MT parameters (Δ*f* = 3 kHz, *B*1 = 10 μT and *D* = 350 ms). A first transverse slice is presented for the three samples (Fig. 5a), zooms corresponding to the red square highlight the structure at the endocardial insertion site of fibers (Fig. 5b). A 3D volume rendering for the three samples is presented, two free-running fibers are segmented for each piece in red (Fig. 5c) at a resolution of 150 µm.

### Histology results

**Fig. 6:**
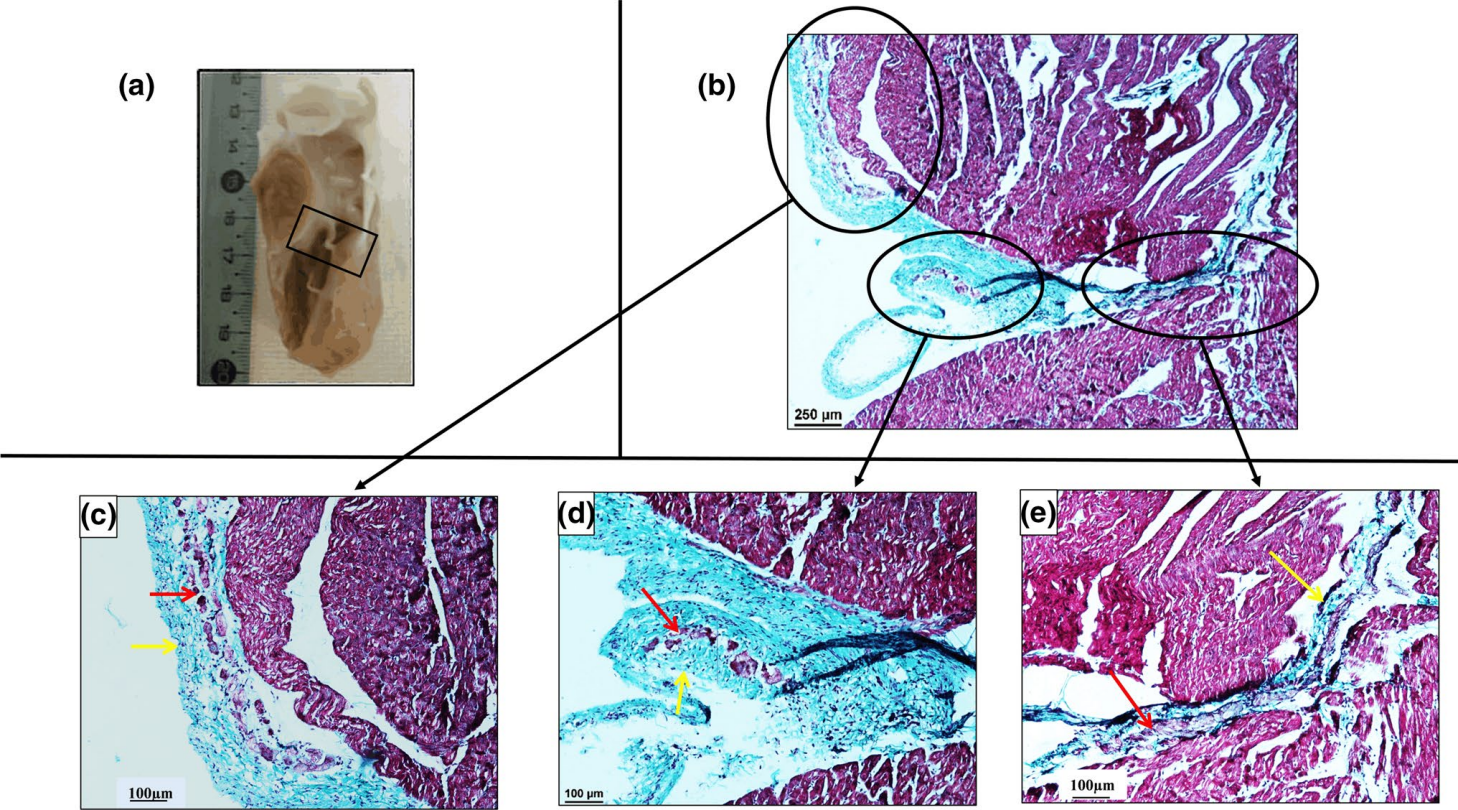
Histology using Masson’s Trichrome stain for a piece of sample 1 (**a**). Microscopy image (**b**) along purkinje fiber. **c** Zoom on different area corresponding to first black circle. **d** Second black circle, (**e**) and third black circle. In blue the collagen (yellow arrow), in pink the cardiomyocytes (red arrow)

Figure 6a and b show typical macroscopic and a histological view, respectively, of the first sample following Masson’s trichrome coloration. Figure 6c–e display zooms to different areas containing PF (black circles in Fig. 6b). A blue–green coloration identifies collagen (yellow arrows) whereas cardiomyocytes appear in pink (red arrows). Figure 6c and d delineate free-running fibers surrounded by a collagen sheath in blue-green. Figure 6e displays insertion of fiber inside the myocardium. The thickness of fibers was found to be 600 µm and 200 µm, respectively. External fibers located close to cardiac muscle displayed a thickness of around ~ 180 µm. It is noted that the thickness of intramural Purkinje fibers is considerably reduced, down to 50–80 µm, and corresponding with the presence of collagen fibers within the cardiac muscle.

### Detection of collagen I and collagen III

**Fig. 7:**
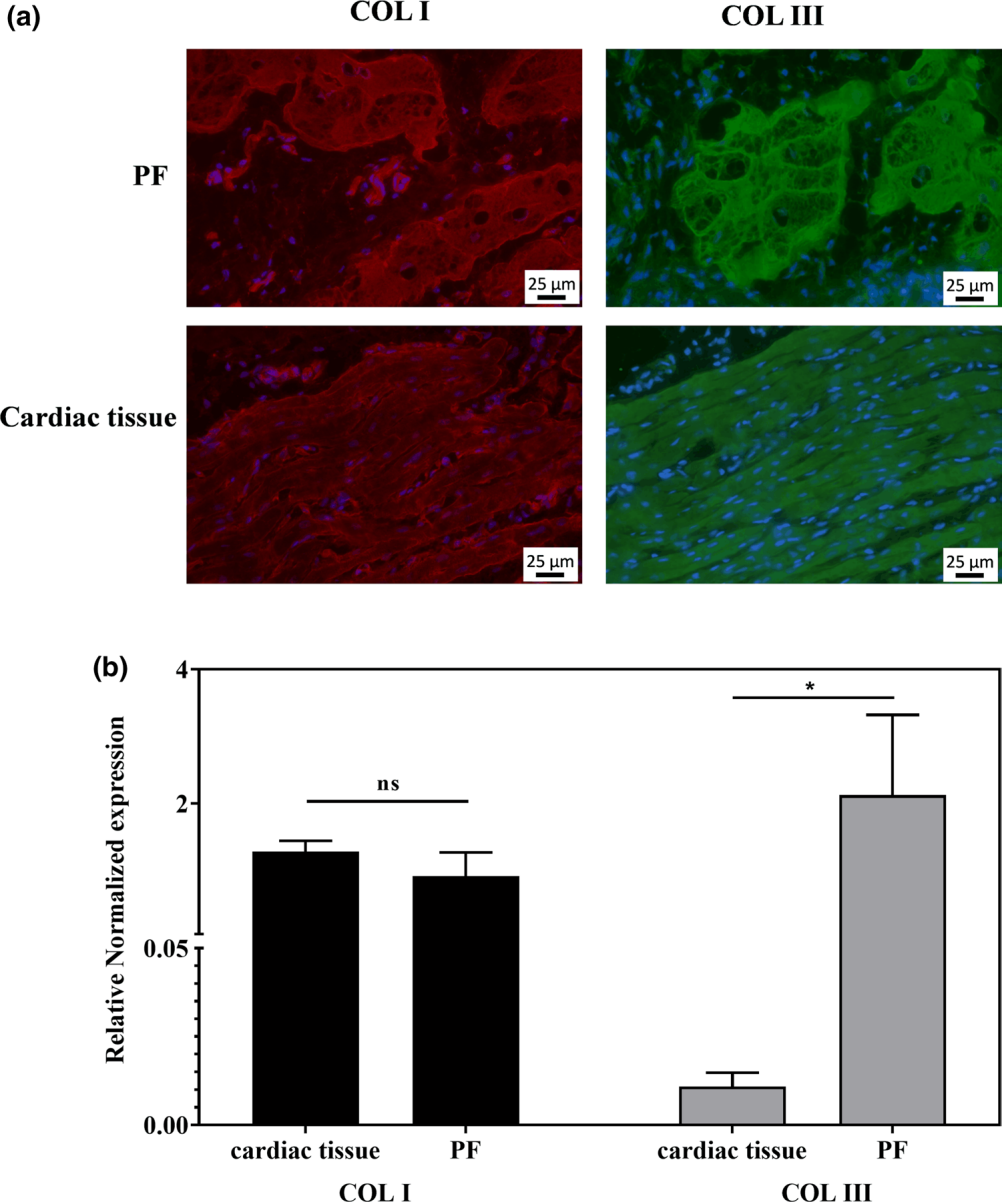
**a** Illustrative examples of immunofluorescence (IF) staining on Purkinje cells (PF) and cardiomyocyte (cardiac tissue) for collagen I (col I in red) and collagen III (col III in green) with DAPI staining in Blue. **b** Bar graphs illustrating quantitative results using q-PCR to evaluate expression of collagen I (col I) and collagen III (col III) in Purkinje fibers (PF) and cardiac tissue

Figure 7a displays illustrative examples resulting from immunofluorescence staining to show response to collagen I and III on PF and cardiac tissues. No difference in intensity for collagen I (in red) is observed between purkinje cells and cardio myocytes whereas for collagen III a more pronounced intensity (in green) is observed for purkinje cells than for cardiomyocytes. Staining with DAPI confirmed presence of cells in blue in tissues for all immunofluorescence images.

Graph bars (in Fig. 7b) present q-PCR expression results for cardiac tissue and PF on collagen I and collagen III. For collagen I, mean values of 1.28 ± 0.16 and 0.92 ± 0.35 are calculated for cardiac tissue and purkinje fibers, respectively, without statistical significant difference. For collagen III, mean values of 0.011 ± 0.004 and 2.12 ± 1.19 were calculated for cardiac tissue and purkinje fibers, respectively, resulting in a significant difference (*p* = 0.044) between both structures.

## Discussion

In this work, we studied MRI parameters at high field (9.4 T) to visualize in 3D the PF network in the heart from large mammalian species. We showed that conventional contrast mechanisms relying on relaxation parameters or proton density result in limited contrasts between PF and myocardium. Relaxation times measured here are similar to those reported in previous studies at 9.4 T on fixed ex vivo rat heart [27–30]. An alternative approach was implemented to improve contrast between PF and myocardium, using MT. Both 2D and 3D acquisitions from three pigs hearts were successfully imaged with high correspondence to collagen-sheathed Purkinje fibers identified by histology.

With MT imaging, we were able to segment PF from cardiac tissue and tendons (red arrow in Fig. 1), whereas very limited contrast (CNR = 0.03 ± 0.01) could be observed between tendons and cardiac tissue (image not shown). We showed that MTR is non-zero for both tissue and fibers. However, a greater sensitivity in myocardium provided contrast with fibers. Since the effect is stronger in tissue, this technique is suitable to increase its contrast relative to fiber. The optimal contrast over the investigated experimental conditions was found at 5 kHz for the highest *B*1 value (15 µT) applied during 450 ms. However, the associated temperature rise was excessive (higher than 3 °C and up to 6 °C) under these experimental conditions. Selecting lower energy deposition by setting a *B*1 of 10 µT at around 3 kHz during 350 ms resulted in similar contrast with much lower temperature increase (less than 2 °C).

### MT and gadolinium contrast agent

In our experiments, we chose to perfuse the heart sample with gadolinium during the fixation process to reduce *T*1 saturation effects. 0.2% of GD Dota was added in the perfusion preparation of samples. This contrast agent dose is a good compromise to obtain an increase of signal-to-noise ratio by decreasing *T*1 values without increasing susceptibility effects on fixed tissue at 9.4 T [31]. In this study, *T*1, *T*2, *T*2* and *M*0 are similar in the conductive fibers and cardiac tissue on samples with contrast agent, we did not observe a difference of relaxation times and contrast (see Table 1), gadolinium is considered uniformly distributed during the fixation process. Same limited contrast was observed in figure S2 on ex vivo cardiac sample using MT MRI without contrast agent preparation [14]. Interestingly, Jones et al.[32] demonstrated on ex vivo hearts that the presence of a paramagnetic contrast agent increases contrast between ischemic and non-ischemic tissue with MT preparation. This was also observed in oncology: contrast agent administration during acquisition combine with MT preparation intensifies contrast when this difference of contrast is already present without MT [33, 34]. Our results with MT are in agreement with those from recent studies using MT to target collagen within fibrosis, such as for imaging progression of renal fibrosis in mice at 16.4 T [20]. The same team applied the MT method on swine kidney in vivo at 3 T with identical methodology, which was further validated using histology [21].

As a result, shorter repetition times could be applied, leading to shorter acquisition duration while preserving high signal-to-noise ratios (> 40) at a 150 µm isotropic spatial resolution whatever the experimental conditions.

### MT and temperature effect

MT protocol using off-resonance pulses requires long acquisition times to deliver sufficient energy to obtain the desired image contrast. However, assuming an acceptable SNR limit of 25 for exploitable image analysis, the number of averages in 3D acquisition could be divided by 4 and consequently decrease the acquisition time further. The repetition time was deliberately chosen with long intervals (> 4 *T*1) to selectively emphasize the effect of MT, with a negligible *T*1 contrast (less than 1% with Gd).

In our 2D acquisition protocol, MT pulses resulted in an accumulated energy deposition of 2.2 ± 0.5 W/TR and non-negligible temperature increase. In 3D, a shorter TR was selected to avoid excessive acquisition durations, but at the cost of an increase of energy deposition of 6.5 ± 1 W/TR. Although this value was acceptable to avoid MR coil alteration (maximal allowed energy deposition of 28 W/TR), the increase in temperature was around 5 °C, which was considered at no risk for tissue integrity since the absolute temperature never exceeded 24 °C. Although *T*1 varies with temperature [35], these variations remain modest and of the order of 1% for an increase of temperature of 1 °C (see Figure S1 in supplementary materials). The effect on the observed contrast here cannot be considered prominent since the flip angle of the sequence was adjusted at the Ernst angle for repetition times of 350 ms (in 3D) and 2000 ms (in 2D) at the beginning of the acquisition.

### MT and collagen content

A decrease in SNR was expected using MT since extracellular scaffold proteins (typically collagen) are present in almost every compartment of cardiac muscle. Yet, the epimysial layer of the extracellular matrix surrounding PF is extensive, particularly in free-running fibers. Consequently, MTR provided differential signal between myocardial tissue and PF-containing regions, resulting in increased contrast. Such a difference could be further related to a variable ratio of collagen types between cardiac muscle and PF. Indeed, we demonstrated with immunofluorescence an intense response to collagen III in PF in comparison to myocardium and we observed a similar response to collagen I for cardiac tissue and conductive fibers. These observations were validated quantitatively with RT-qPCR, showing an intense positive reaction of collagen III in fibers in comparison to cardiac tissue. These results are in good agreement those reported in the literature for the myocardium, where type I collagen was reported to be the most abundant [25], whereas immunochemistry performed on sheep cardiac conductive fibers [2] showed that connective sheath displays a moderately positive reaction for type I but an intense positive reaction for type III of collagen. At 11.7 T, Qiao et al. [26] measured on ex vivo human carotid samples a MTR of 54 ± 9% for collagen type I, whereas this value dropped to 11 ± 6% for type III. Such a difference is thus expected to result in an increased contrast between these structures, despite both of them exhibiting MT.

### MT and fixation preparation

The process of tissue fixation may also influence MTR values measured ex vivo in comparison to in vivo data, as reported by Fishbein et al. [36] at 9.4 T. They reported a slight increase of MTR (12.5%) after fixation (with identical formalin concentrations), which cannot explain the MTR values observed in our experiments (25–44%, see Table 2).

Working on ex vivo samples has an impact on *T*2 and *T*1 with reduced values in comparison to in vivo or unfixed post mortem samples [37]. Due to reduction of *T*1 and *T*2 and diffusivity, DTI acquisition at an isotropic resolution of 150 µm remained challenging. The most important inconvenient with DTI is the long acquisition time to obtain a resolution in 3D of 150 µm with sufficient SNR. It has been reported on fixed ex vivo sample that *T*1 and *T*2 contrast can be increased by a prolonged washing of the sample (wash out fixative and rehydrate the sample) before scanning in saline or phosphate‐buffered saline (PBS) [38]. This preparation before MRI scanning has not been investigated here but could improve our acquisition protocol.

The increase in contrast observed in the present work using the MT technique can reasonably be attributed to different collagen types in myocardium and PF, due to different content of collagen I and collagen III demonstrated by immunofluorescence staining and q- PCR.

Histological data are in good agreement with previously published results. Pope et al. [39] performed 3D acquisition of a small piece of rabbit heart with a voxel resolution of 1 µm^3^ using confocal microscopy to follow the collagen network outside and inside the muscle. Ono et al. [40] demonstrated that conductive fiber network varies between species. Indeed, in goats and sheep Purkinje strands consisting of 2–8 oval cells, they demonstrated that the mesh of the PF network varied between 200 and 1500 µm using light electron microscopy. Histological images in the present study showed fibers with cardiomyocytes surrounded by collagen, which are characteristic of the free PF network, as described in the literature [2, 41]. A transitional structure at the junction between free-running PF and myocardium can be observed (Fig. 6). Tranum-Jensen et al. [42] observed the same anatomic arrangement at the connection of PF to the cardiac muscle with a large PF fibers connected via thin branches to the ventricular mass.

The main limitation of this study remains the spatial resolution. Although we performed acquisition at an isotropic resolution of 150 µm, allowing to follow free-running PF in pig hearts (~ size of human heart), such a voxel size remains at the limit to track intramural PFs, where a resolution of 100 µm or better would be required. At this resolution, the resulting SNR would be divided by 3, leading to resulting SNR of around 10 (at the limit for interpretation) with our experimental setup. The use of higher averaging of smaller samples with dedicated instrumentation (smaller coil combined with a cryoprobe for example) in combination with further image processing is amenable to provide exploitable data at such resolution. In the present work, we chose to focus the analysis of MT parameters to increase contrast, at the cost of a compromise on image resolution. Alternatively, MT contrast could also be generated with other pulses design [43, 44] or acquisition techniques such as on-resonance MT [45] to reduce total acquisition duration, energy deposition, and temperature increase. This was out of the scope of the present work.

In the future, the MT methodology in ex vivo cardiac samples could also be applied to pathological hearts [45] with regions of infarct to evaluate contrast obtained between lesion sites (with collagen remodeling) and healthy regions to support realistic computational simulations and to understand electrophysiology and arrhythmias disorders [10, 39].

We demonstrated the added value of the MT technique to improve contrast between PF and cardiac muscle. Although all tissues exhibited a reduction in SNR in presence of MT, the contrast between PF and the cardiac tissue has been increased by more than 50% as compared to standard proton density weighted sequence. 3D high-resolution images at 150 µm are feasible and allow to visualize the free-running PF and their connection within the myocardium.

## Supplementary Information

Below is the link to the electronic supplementary material.

**Figure S1: **Correlation between temperature (°C) and relaxation times *T*1 (ms) measured. Line represents linear fits through the datapoints (TIF 546 KB)

**Figure S2: **Results obtained for a sample without gadolinium. **a** Photographs of ex vivo sample without contrast agent after fixation. Black box indicate the fiber of interest in the sample. **b** Mean of MR parameters *T*1, *T*2 and *T*2* (ms) are calculated and M0 (a.u) measured for both cardiac tissue and fiber in the sample. **c** 2D images obtained without the preparation module (figure on the left) and using MT (figure in the right). Red arrow indicate the conductive fiber (TIF 3108 KB)

## Abbreviations

PF: Purkinje fibers
MT: Magnetization transfer
MTR: Magnetization transfer ratio
