# Supplementary figures and images for: 3D magnetization transfer (MT) for the visualization of cardiac free-running Purkinje fibers: an ex vivo proof of concept
Source: MAGMA. 2021 Jan 23;34(4):605–18. doi: 10.1007/s10334-020-00905-w (PMC8338918; doi:10.1007/s10334-020-00905-w)

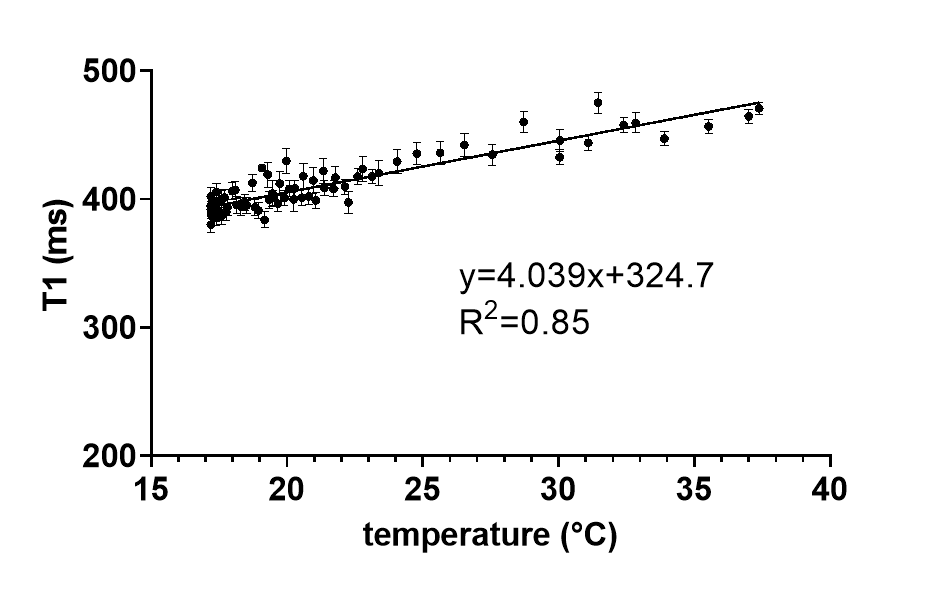

Supplement: Supplementary file 1 — Figure S1: Correlation between temperature (°C) and relaxation times T1 (ms) measured. Line represents linear fits through the datapoints (TIF 546 KB) [file 10334_2020_905_MOESM1_ESM.tif]
